# The TAAR1 antagonist EPPTB ameliorates colitis via serotonin inhibition

**DOI:** 10.1016/j.bbrep.2025.102432

**Published:** 2026-01-06

**Authors:** Leyi Luo, Tong Zhang, Linlin Liang, Weixin Chen, Tao Wang, Wenke Chen, Haitao Xiao, Guangtao Zhang

**Affiliations:** a Department of Hepatobiliary and Pancreatic Surgery, Peking University Shenzhen Hospital, Shenzhen, Guangdong, 518036, China; b Guangdong Provincial Key Laboratory of Chinese Medicine Ingredients and Gut Microbiomics, School of Pharmacy, Shenzhen University Medical School, Shenzhen University, Shenzhen, Guangdong, 518055, China; c Department of Gastroenterology, Peking University Shenzhen Hospital, Shenzhen, Guangdong, 518036, China; d School of Pharmacy, Guizhou Medical University, Guiyang, 550014, China

**Keywords:** IBD, Trace amines, TAAR1, EPPTB, 5-HT

## Abstract

Inflammatory bowel disease (IBD) is characterized by gut dysbiosis and impaired microbial metabolite signaling. Emerging evidence suggests that gut microbial metabolites, such as trace amines (tryptamine, phenethylamine, and tyramine), function as endogenous TAAR1 agonists and may contribute to IBD pathogenesis. Our study identified elevated fecal trace amine levels in ulcerative colitis (UC) patients and DSS-induced colitis mice. In vitro, exposure to these trace amines enhanced 5-HT secretion in QGP-1 cells and ex vivo mouse colonic tissues, and this effect could be blocked by the TAAR1 antagonist EPPTB. In vivo, EPPTB treatment significantly mitigated DSS-induced colitis, as demonstrated by reduced weight loss, improved disease activity index (DAI), preserved colon length, and attenuated histopathological damage. Moreover, TAAR1 blockade reduced pro-inflammatory cytokines (TNF-α, IL-6, IL-1β) and increased IκB-α expression, restored intestinal barrier integrity (upregulating occludin and ZO-1, while downregulating cclaudin-2), and lowered colonic 5-HT levels by suppressing TPH1 expression. These findings suggest that TAAR1 inhibition alleviates colitis by modulating 5-HT signaling, positioning it as a promising therapeutic target for IBD.

## Highlights

• TAAR1 antagonism suppresses trace amine-stimulated 5-HT secretion in QGP-1 cells and ex vivo mouse colonic tissues.
• TAAR1 inhibition alleviates colitis severity, intestinal inflammation, and barrier dysfunction.
• TAAR1 antagonism reduces colonic 5-HT content through transcriptional downregulation of TPH1.
• Targeting TAAR1 presents a promising new therapeutic paradigm for IBD management.

## 1 Introduction

Modern lifestyles—characterized by chronic stress, erratic schedules, and poor dietary habits—are driving a rise in digestive disorders. Among these, inflammatory bowel disease (IBD), including ulcerative colitis (UC) and Crohn's disease (CD), stands out as a debilitating chronic condition marked by persistent inflammation, recurrent flare-ups, and limited treatment efficacy. Due to its challenging management and often unpredictable course, IBD is widely regarded as a refractory condition [1,2]. The exact cause of IBD remains unknown. Current therapies focus on inducing and maintaining remission using aminosalicylates, corticosteroids, and immunosuppressants. However, these drugs often offer only short-term relief and can cause significant adverse effects [3,4]. Biologics have emerged as the frontline treatment, yet approximately 30 % of patients fail to respond initially, and nearly half of responders lose effectiveness within five years. Moreover, these drugs carry risks of neurological and liver complications and come with a high financial burden [[5], [6], [7], [8], [9], [10]]. Clearly, more effective treatment options are urgently needed.

IBD arises from a complex interplay of genetic predisposition, environmental triggers, immune dysregulation, and gut microbiome imbalances. Recent studies highlight that gut microbial metabolites—particularly trace amines (such as tryptamine, phenethylamine, and tyramine)—act as endogenous TAAR1 agonists. These have been shown to promote serotonin (5-HT) release from enterochromaffin cells by activating TAAR1 [11,12]. Notably, the gut is the source of 90 % of the body's 5-HT [13]. In the context of IBD, 5-HT has a dual role in exacerbating the disease: 1) It binds to 5-HT2A/3/7 receptors, activating immune cells (such as neutrophils and T cells) that release inflammatory substances damaging the intestinal lining [14]. 2) It affects bowel movements and secretions, leading to common IBD symptoms like diarrhea [15]. Based on these findings, we hypothesize that TAAR1 may represent a novel therapeutic target for IBD. This study aims to evaluate the effects of the TAAR1 antagonist EPPTB in a DSS-induced colitis model, focusing on inflammation, barrier function, and 5-HT signaling, to explore its therapeutic potential.

## 2 Materials and methods

### 2.1 Culture and treatment of QGP-1 cells

The human pancreatic endocrine cell line QGP-1, obtained from COBIOER Biological Company Co., Ltd. (Nanjing, China), was maintained in RPMI 1640 medium containing 10 % fetal bovine serum (FBS), 100 U/mL penicillin, and 100 μg/mL streptomycin, under a humidified atmosphere of 5 % CO_2_ at 37 °C. QGP-1 is known to synthesize 5-HT. To investigate the effects of tryptamine (Tryp), phenylethylamine (PEA), and tyramine (Tyr) on 5-HT production, the cells were treated with or without 20 μM of each amine for 24 h, based on our previous experimental data [11]. Furthermore, to assess the role of TAAR1 in amine-induced 5-HT production, the TAAR1 inhibitor EPPTB was applied at a concentration of 20 μM.

### 2.2 Human fecal samples

Fecal samples were collected from 20 patients with active ulcerative colitis (UC) and 15 healthy controls recruited from the Department of Gastroenterology, Peking University Shenzhen Hospital (a teaching hospital of Shenzhen University), between January 1 and August 30, 2024. The diagnosis of active UC was confirmed by a comprehensive assessment of clinical symptoms, endoscopic findings, and histological examination. All participants provided written informed consent. The study protocol was approved by the Institutional Review Board for Medical Ethics of Shenzhen University (Approval No. PN-202200128). Samples were snap-frozen and stored at −80 °C.

Exclusion criteria comprised recent (within 3 months) use of antibiotics, steroids, or probiotics; intestinal infection; functional gastrointestinal disorders; short bowel syndrome or prior gastrointestinal surgery; malignancy; diabetes; pregnancy; systemic inflammation; active cardiovascular, renal, or hepatic disease; and autoimmune conditions. Healthy controls had not used antibiotics or probiotics in the past month. General characteristics of participants are summarized in Supplementary Table S1.

### 2.3 Animals

Male C57BL/6 mice (6–8 weeks old, weighing 20–25 g) were procured from Beijing Vital River Laboratory Animal Technology Co., Ltd. (Beijing, China; License No. SCXK (Jing) 2021-0006). The animals were housed in the specific pathogen-free facility of Shenzhen University Medical School under standard conditions, including a 12-h light/dark cycle, controlled ambient temperature, and ad libitum access to food and water. All experimental protocols were approved by the Animal Ethics Committee of Shenzhen University (Approval No. IACUC-202400006) and conducted in strict accordance with institutional and national guidelines for the care and use of laboratory animals.

### 2.4 Ex vivo colonic tissues study

To evaluate the effects of PEA, Tryp and Tyr on colonic 5-HT levels, the mouse colon tissues were sectioned into 0.5 cm segments and transferred to a 12-well cell culture plate. The explants were immersed in DMEM/F12 medium supplemented with 5 % fetal bovine serum, 1 × penicillin-streptomycin, and 20 μg/mL gentamycin, and then treated with the respective amines and EPPTB as specified. Cultures were maintained at 37 °C under an atmosphere of 5 % CO_2_ and 95 % air. At designated time points, 200 μL of medium was sampled from each well for subsequent 5-HT quantification. Subsequently, the remaining colon segments were homogenized in ice-cold RIPA buffer supplemented with protease and phosphatase inhibitors for protein quantification using a BCA assay kit (Beyotime, Shanghai, China).

### 2.5 Chronic colitis model and experimental design

A chronic colitis model was induced by dextran sulfate sodium (DSS) administration with minor modifications from our previously established protocol [16]. Briefly, DSS was dissolved in drinking water at a concentration of 1.8 % (w/v). Each induction cycle comprised five days of 1.8 % DSS exposure followed by seven days of regular drinking water. The experimental timeline spanned from day 13 to day 29 (final day). Following one cycle of DSS administration, all mice developed consistent colitis and were randomly allocated into two groups (n = 6 per group): DSS alone, and DSS plus EPPTB (10 mg/kg; MedChemExpress, New Jersey, USA). Both groups exhibited comparable baseline colitis severity and similar daily water consumption. EPPTB was freshly prepared each day and administered orally in a 400 μL volume. A normal control group (n = 6) received filtered drinking water throughout the study period. Body weight and disease activity index (DAI) were monitored daily, with DAI scores calculated based on weight loss, stool consistency, and fecal occult blood as previously described [17].

### 2.6 Intestinal permeability evaluation

Intestinal permeability was evaluated in vivo using fluorescein isothiocyanate–dextran (FITC-dextran; 4 kDa; Sigma-Aldrich, USA) according to established protocols [3]. Briefly, colitis-induced mice were fasted for 12 h and subsequently administered FITC-dextran (0.6 mg/g body weight) via oral gavage prior to experimental termination. Three hours post-administration, mice were anesthetized, and blood samples were collected via the retro-orbital plexus for fluorescence spectrophotometric quantification of serum FITC-dextran levels.

### 2.7 Histopathological evaluation

At the endpoint, mice were euthanized, and colon length was measured as a macroscopic indicator of inflammation. Colon tissues were fixed in 4 % paraformaldehyde (PFA), paraffin-embedded, sectioned (4–5 μm), and stained with hematoxylin and eosin (H&E). Blinded histopathological scoring was performed following our established protocol [18].

### 2.8 Quantification of PEA, Tryp, Tyr and 5-HT

Fecal extracts, colon tissues, and cell supernatants were processed with methanol containing an internal standard solution (600 nM tryptophan-d5), centrifuged (14,000 rpm, 15 min, 4 °C), and analyzed via UHPLC-QQQ-MS (Agilent 6460). Chromatographic separation used a Waters BEH C18 column (2.1 × 50 mm, 1.7 μm) with a gradient elution (0.1 % formic acid in water [A] and acetonitrile [B]) at 1 mL/min. The gradient program was: 2 % B (0–0.5 min), 2–30 % B (0.5–4 min), 30–100 % B (4–6 min), 100 % B (6–8 min), and re-equilibration at 2 % B (8.1–10 min). The MS analysis was performed with a quadrupole time-of-flight (Q-TOF) tandem mass spectrometer (Agilent Technologies, Santa Clara, CA, USA) equipped with electrospray ionization (ESI) in negative ionization mode, as described in our previous study [16].

### 2.9 RNA extraction and qPCR

Total RNA was extracted from colon tissues with TRIzol® Reagent (Thermo Fisher Scientific, USA), and complementary DNA (cDNA) was reverse-transcribed using a commercial kit (TaKaRa, Shiga, Japan). Quantitative real-time PCR (qPCR) was subsequently performed with SYBR Green Master Mix (Roche Diagnostics GmbH, Mannheim, Germany) on an ABI 7500 Real-Time PCR System. All mRNA expression data were normalized to the endogenous control GAPDH, and corresponding primer sequences are listed in Supplementary Table S2.

### 2.10 Cytokine measurement (ELISA)

The total protein content of colon tissue homogenates was first determined using a BCA protein assay kit (Beyotime, Shanghai, China). Subsequently, the concentrations of cytokines (TNF-α, IL-1β, IL-6, and IL-10) were measured with commercial kits (JiangLai Biology, Shanghai, China) in accordance with the manufacturer's protocols. All tissues were homogenized in protein extraction buffer containing protease and phosphatase inhibitors.

### 2.11 Western blot detection

Western blot analysis was conducted according to established methodology [3]. Briefly, following protein quantification, lysates from colon tissues were resolved on SDS-polyacrylamide gels and electrophoretically transferred onto nitrocellulose membranes. The membranes were subsequently blocked with 5 % bovine serum albumin (BSA) and probed with specific primary antibodies, followed by incubation with appropriate horseradish peroxidase (HRP)-conjugated secondary antibodies. Protein bands were visualized with an enhanced chemiluminescence (ECL) substrate (BIO-RAD, 1705061, Hercules, CA, USA), and band intensities were quantified using ImageJ software (National Institutes of Health, Bethesda, MD, USA). The following primary antibodies, all diluted at 1:1000, were employed: GAPDH (Cell Signaling Technology, 2118S), IκB-α (Cell Signaling Technology, 9242), occludin (Cell Signaling Technology, 91131), and claudin-2 (Cell Signaling Technology, 48120).

### 2.12 Statistical analysis

Data are presented as mean ± SD. All statistical analyses were performed using GraphPad Prism 8, with a *P*-value <0.05 considered statistically significant. Comparisons between two groups were analyzed by Student's t-test. For comparisons involving three or more groups, one-way ANOVA was employed, followed by Dunnett's test for comparisons against a single control group or by Tukey's test for all pairwise comparisons.

## 3 Results

### 3.1 Alterations in fecal levels of PEA, Tryp, and Tyr in UC patients and DSS-induced colitis mice

**Fig. 1:**
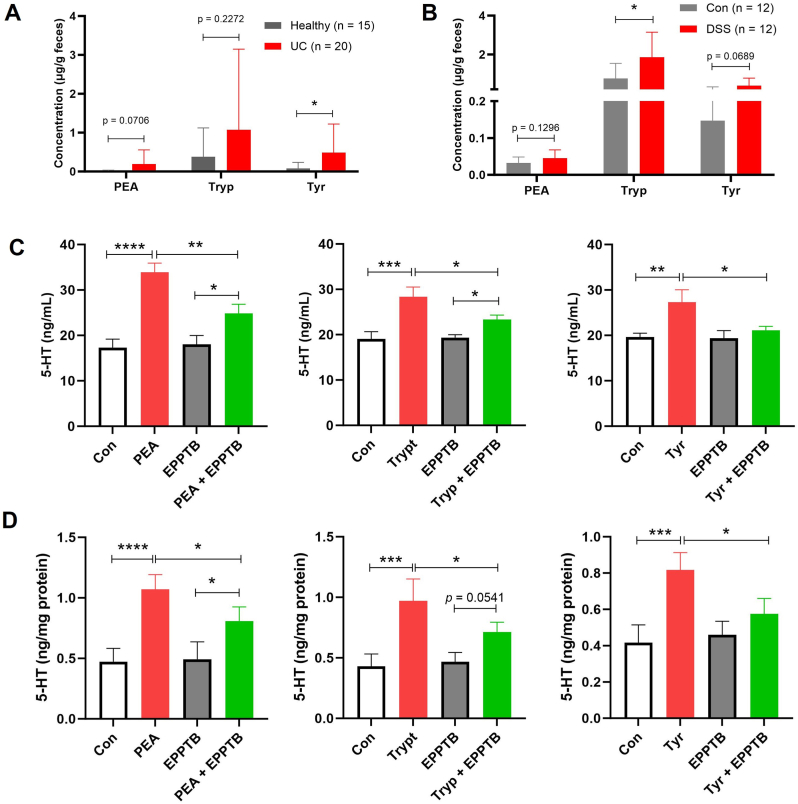
**Fecal Tryp, PEA, and Tyr in UC and DSS-colitis mice, and TAAR1 inhibition attenuates their 5-HT release from QGP-1 cells and mouse ex vivo colonic tissues.** (A) Fecal levels of Tryp, PEA, and Tyr in healthy control (n = 15) and UC patients (n = 20). (B) Fecal levels of Tryp, PEA, and Tyr in Control (Con) mice and DSS-induced colitis mice (n = 12). Student's t-test were used to determine differences between two groups in Data A and B. (C) The 5-HT levels in QGP-1 cells following 24-h treatments with PEA, Tryp, or Tyr (20 μM) alone, and each amine in combination with EPPTB (20 μM) (n = 3). (D) The 5-HT levels in mouse ex vivo colonic tissues following 2-h treatments with PEA, Tryp, or Tyr (50 μM) alone, or each amine in combination with EPPTB (50 μM) (n = 4). One-way ANOVA followed by Tukey's multiple comparisons test was used to determine differences between two groups in Data C and D. Data are presented as means ± SD. *P < 0.05, **P < 0.01, ***P < 0.001 and ****P < 0.0001.

Fecal concentrations of PEA, Tryp, and Tyr were analyzed in UC patients and DSS-induced colitis mice. As shown in Fig. 1A, UC patients exhibited elevated levels of these trace amines compared to healthy volunteers, with Tyr showing a statistically significant increase. Similarly, Fig. 1B demonstrates that DSS-induced colitis mice also had higher fecal concentrations of PEA, Tryp, and Tyr relative to control mice, with Tryp reaching statistical significance. These findings collectively indicate a trend of increased fecal levels of PEA, Tryp, and Tyr in both clinical and experimental colitis, although the specific amines that reached statistical significance differed between species.

### 3.2 TAAR1 blockade attenuates PEA-, Tryp-, and Tyr-induced 5-HT release

Based on previous findings from our group demonstrating that Tryp and PEA promote 5-HT release [11], we hypothesized a potential involvement of TAAR1 signaling, given the established role of these amines as TAAR1 agonists. To test this, we employed the selective TAAR1 antagonist EPPTB in both QGP-1 cells and ex vivo mouse colonic cultures. Pharmacological blockade of TAAR1 with EPPTB significantly inhibited the 5-HT release induced by all three trace amines (Fig. 1C and D).

### 3.3 TAAR1 inhibition alleviates disease severity in DSS-induced colitis

**Fig. 2:**
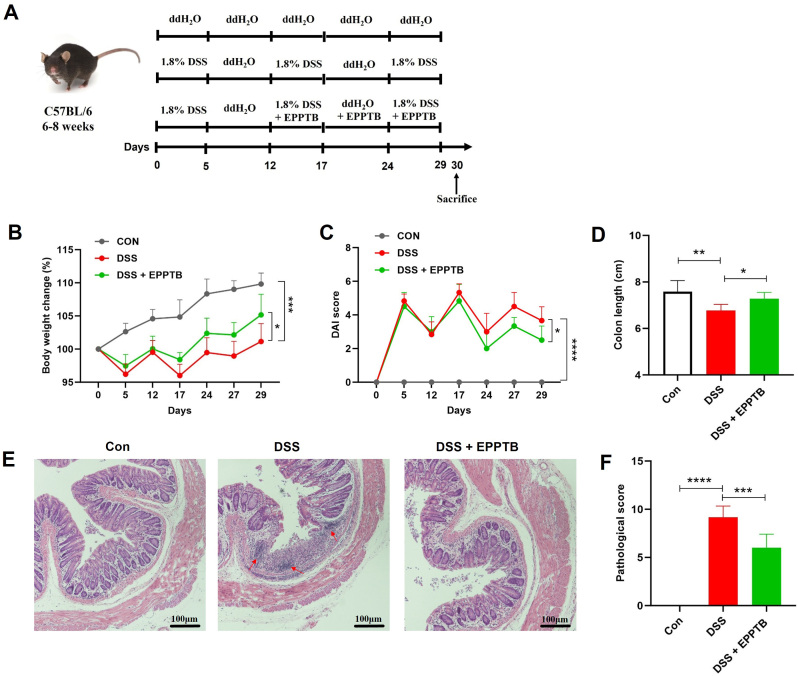
**TAAR1 inhibition alleviates disease severity in DSS-induced colitis.** (A) Schematic diagram of the experimental procedure. (B) The time-dependent changes in body weight. (C) The time-dependent changes in disease activity index (DAI). (D) Colon length. (E and F) H&E staining ( × 200, scale bar = 100 μm) and pathological scores (red arrows: immune cell infiltration). One-way ANOVA followed by Dunnett's test was used to determine differences among the three groups. Data are presented as means ± SD (n = 6). *P < 0.05, **P < 0.01, ***P < 0.001 and ****P < 0.0001.

Given the detrimental role of 5-HT in IBD and the elevated levels of TAAR1-activating amines (Tryp, PEA, and Tyr) in both UC and DSS-colitis models, we evaluated the therapeutic potential of TAAR1 inhibition in chronic DSS-colitis (Fig. 2A). Oral treatment with EPPTB substantially alleviated disease severity, as demonstrated by a significant attenuation of body weight loss and a lower DAI score—a composite measure evaluating weight loss, rectal bleeding, and stool consistency (Fig. 2B and C). Furthermore, EPPTB administration counteracted DSS-induced colon shortening (Fig. 2D). Histological analysis revealed that EPPTB reduced colonic tissue damage, including crypt destruction and immune cell infiltration (Fig. 2E), which was corroborated by a significant decrease in the histopathological score relative to the DSS model group (Fig. 2F).

### 3.4 TAAR1 blockade suppresses colonic inflammation in DSS-induced colitis

**Fig. 3:**
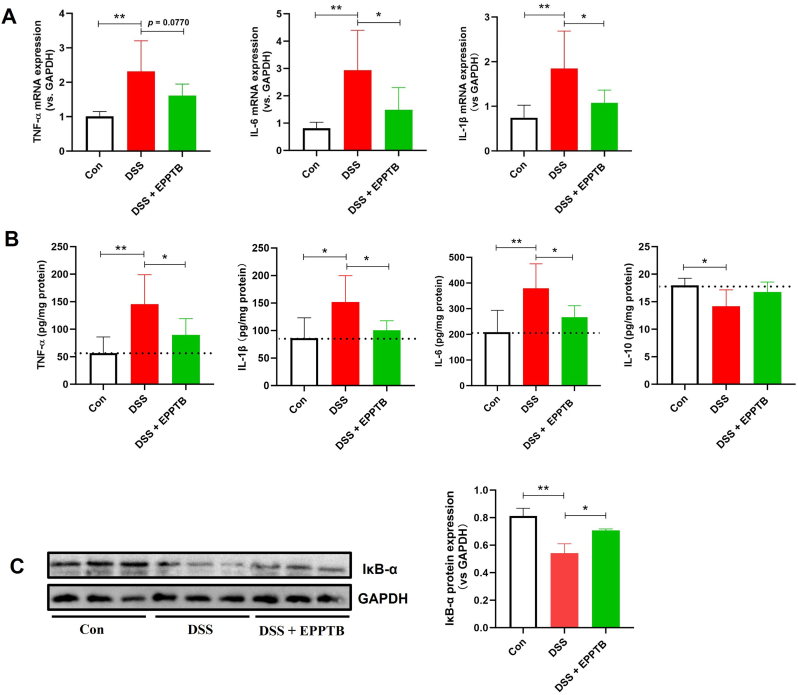
**TAAR1 blockade suppresses colonic inflammation in DSS-induced colitis.** (A) mRNA levels of pro-inflammatory cytokines TNF-α, IL-6, and IL-1β in colon tissues of colitis mice. (B) Protein levels of pro-inflammatory cytokines TNF-α, IL-6, IL-1β and anti-inflammatory cytokine IL-10 in colon tissues of colitis mice. (C) Protein expression of IκB-α in colon tissues of colitis mice. One-way ANOVA followed by Dunnett's test was used to determine differences among the three groups. Data are presented as means ± SD (n = 6 for data A and B; n = 3 for data C). *P < 0.05 and **P < 0.01.

To further investigate the anti-inflammatory effects of TAAR1 inhibition, we assessed the impact of its antagonist EPPTB on colonic cytokines in DSS-induced colitis mice. TAAR1 blockade significantly downregulated the mRNA expression of pro-inflammatory cytokines (TNF-α, IL-6, IL-1β), as measured by qPCR (Fig. 3A). This transcriptional suppression was consistent with a significant reduction in the corresponding protein levels, as determined by ELISA (Fig. 3B). Conversely, protein levels of the anti-inflammatory cytokine IL-10 showed a non-significant increasing trend upon TAAR1 inhibition. Furthermore, Western blot analysis confirmed that EPPTB treatment up-regulated IκB-α, an endogenous inhibitor of the NF-κB pathway, in colonic tissues (Fig. 3C).

### 3.5 TAAR1 blockade improves intestinal barrier integrity in DSS-induced colitis

**Fig. 4:**
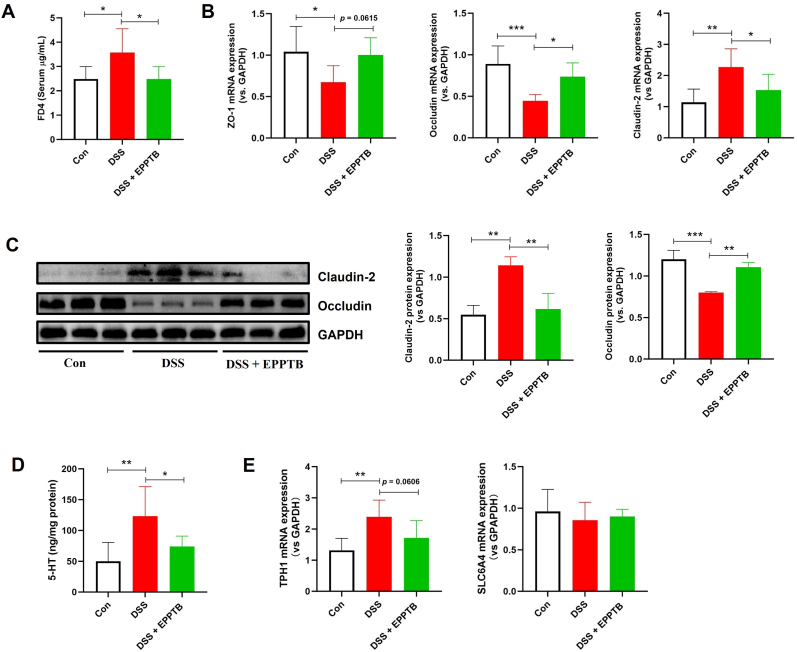
**TAAR1 blockade improves gut barrier integrity and reduces colonic 5-HT in DSS-colitis.** (A) Concentration of FD4 in the blood. (B) mRNA levels of tight junction ZO-1, occludin, and claudin-2; (C) Protein expression of occludin and claudin-2. (D) Concentration of 5-HT in colon tissues of colitis mice. (E) mRNA levels of TPH1 and SLC6A4 in colon tissues of colitis mice. One-way ANOVA followed by Dunnett's test was used to determine differences among the three groups. Data are presented as means ± SD (n = 6 for data A, B, D and E; n = 3 for data C). *P < 0.05, **P < 0.01 and ***P < 0.001.

We next explored whether TAAR1 inhibition preserves intestinal barrier function in DSS-colitis. EPPTB treatment significantly attenuated the increase in gut permeability to FITC-dextran (FD4) (Fig. 4A). At the molecular level, TAAR1 inhibition markedly enhanced the mRNA expression of the tight junction components ZO-1 and occludin (Fig. 4B), and consistently increased occludin protein levels (Fig. 4C). In contrast, EPPTB administration substantially downregulated claudin-2, a pore-forming tight junction protein known to increase permeability, at both the mRNA and protein levels (Fig. 4B and C).

### 3.6 TAAR1 inhibition reduces intestinal 5-HT levels in DSS-induced colitis

To determine the effect of TAAR1 blockade on 5-HT production and metabolism, we analyzed 5-HT levels and related enzyme expression in colon tissues. EPPTB treatment led to a significant decrease in colonic 5-HT content and reduced mRNA expression of TPH1, a key enzyme in 5-HT synthesis (Fig. 4D and E). In contrast, the mRNA expression of SLC6A4 (encoding the serotonin transporter SERT) remained unchanged (Fig. 4E).

## 4 Discussion

This study confirms marked elevations in fecal TAAR1-agonist amines (tryptamine, phenethylamine, tyramine) in both UC patients and DSS-induced colitis mice. Treatment with the selective TAAR1 antagonist EPPTB substantially improved colitis symptoms, including reduced weight loss, lower DAI scores, prevention of colon shortening, diminished tissue damage, and decreased expression of pro-inflammatory cytokines (TNF-α, IL-6, and IL-1β) in colonic tissue. Additionally, TAAR1 inhibition enhanced the intestinal barrier by upregulating tight junction proteins (ZO-1, occludin) and downregulating Claudin-2, while also reducing colonic 5-HT levels and TPH1 expression.

The role of gut dysbiosis in IBD pathogenesis is well-documented, with extensive evidence linking microbial imbalance to immune dysregulation, impaired barrier function, and metabolic disturbances. Multiple studies have consistently reported an increased abundance of certain gut bacteria, such as *E. faecium* and *R. gnavus*, in IBD patients, with levels correlating positively with disease severity [[19], [20], [21]]. Among microbial metabolites, aromatic amines—particularly Tryp, PEA, and Tyr—have emerged as key functional mediators. These compounds, produced by bacteria such as *Enterococcus faecalis, Bacteroides fragilis, Escherichia coli, Providencia rettgeri, and R. gnavus*, disrupt intestinal homeostasis through diverse receptor pathways. For instance, Li et al. demonstrated that gut-derived Tyr suppresses intestinal stem cell (ISC) proliferation via α2A-adrenergic receptor activation, impairing epithelial regeneration and exacerbating DSS-induced colitis [22]. Similarly, Bhattarai et al. found that microbial Tryp enhances colonic epithelial ion transport through 5-HT4 receptor activation, increasing fluid secretion in organoids and accelerating intestinal motility—contributing to diarrhea [23]. Furthermore, Pretorius L and Smith C reported that excessive levels of Tryp, PEA, and Tyr can exacerbate gastrointestinal inflammation, with Tyr exhibiting the most pronounced detrimental effects [24]. Consistent with this, our prior work further showed that Tryp, PEA, and Tyr promote 5-HT release, establishing a neuro-epithelial feedback loop that amplifies intestinal hypermotility [11]. Collectively, these findings strongly suggest that aromatic amines play a critical role in IBD progression. Our current observation of elevated fecal Tryp, PEA, and Tyr levels in UC patients and DSS-colitis mice aligns with previous reports in IBD patients and animal models [16,22,25], further supporting their involvement in disease pathogenesis.

TAAR1 (trace amine-associated receptor 1), a G protein-coupled receptor (GPCR), is widely expressed in the central nervous system (CNS) and peripheral tissues. In the CNS, it localizes to monoaminergic neuron-rich regions, including the ventral tegmental area (VTA), substantia nigra pars compacta (SNc), striatum, and prefrontal cortex (PFC). Peripherally, TAAR1 is found in the pancreas, gastrointestinal tract, and immune cells, where it modulates metabolic and immune responses. Functionally, TAAR1 couples with Gs proteins to activate adenylate cyclase (AC), increasing cAMP levels and stimulating PKA signaling, while also regulating ERK/Akt activity via β-arrestin-dependent pathways—mechanisms critical for neuroplasticity and cell survival. Recent work by Vaganova et al. [26] using GEO database analysis revealed high TAAR1 expression in mouse intestinal epithelial cells, enteroendocrine cells, villous cells, and myenteric neurons, suggesting a role in enteroendocrine cell maturation. Our studies further identified TAAR1 on enterochromaffin cells, where microbial trace amines (Tryp, PEA, and Tyr) activate the receptor to trigger 5-HT release—a mechanism implicated in diarrhea in irritable bowel syndrome (IBS) patients [11]. In IBD, TAAR1 also modulates macrophage activity and inflammation. Bugda Gwilt et al. [27] reported that TAAR1 blockade significantly attenuates Tyr-induced proinflammatory cytokine production in bone marrow-derived macrophages. Additionally, the chemokine CCL5, a key player in intestinal inflammation, is regulated by TAAR1 in T cells; siRNA-mediated TAAR1 knockdown reduced PEA-induced CCL5 expression [28]. Based on these findings, EPPTB appears to alleviate colitis through a dual mechanism: directly inhibiting 5-HT release from enterochromaffin cells, while simultaneously modulating immune cell activity, thereby collectively attenuating intestinal inflammation. Although the present study did not directly evaluate the correlation between fecal trace amine levels and disease severity, existing literature suggests that such metabolites hold potential clinical discriminative value in IBD patients [22,29,30]. Future investigations may further explore their utility as biomarkers.

Despite the compelling evidence provided by this study, several limitations warrant careful consideration. First, the limited sample size precludes definitive conclusions, necessitating an expanded clinical cohort to more conclusively define the dynamic changes in trace amine levels in UC patients and their correlation with disease severity. Second, the DSS-induced colitis model only partially recapitulates the complex pathophysiology of human inflammatory bowel disease, underscoring the need for future validation using spontaneous models such as IL-10 knockout mice and human intestinal organoid systems. Third, potential central nervous system-related side effects of EPPTB remain unexamined; developing gut-restricted TAAR1 antagonists or non-absorbable compounds could help minimize off-target effects and enhance therapeutic safety. Furthermore, the precise mechanism of action of EPPTB remains elusive—specifically, whether it directly modulates microbial composition or indirectly influences host perception of microbial metabolites represents a critical question requiring further investigation. Finally, employing TAAR1 knockout models or alternative TAAR1 antagonists for cross-validation in subsequent studies would strengthen the evidence for TAAR1-specific involvement.

From a translational medicine perspective, targeting TAAR1 represents a promising novel therapeutic strategy for IBD. Beyond TAAR1, other TAAR subtypes (e.g., TAAR5, TAAR8, TAAR9) and their ligands (such as trimethylamine, cadaverine, and putrescine) also demonstrate dysregulated expression or metabolic disturbances in IBD patients [31], suggesting a broader involvement of the TAAR family in IBD pathogenesis. The future development of gut-restricted TAAR1 antagonists, or combination therapies simultaneously targeting multiple TAAR signaling pathways, may offer more precise and safer treatment options for IBD patients.

## 5 Conclusions

In summary, our study demonstrates that microbiota-derived endogenous TAAR1 agonists Tryp, PEA, and Tyr are elevated in both ulcerative colitis patients and DSS-induced colitis mice. Pharmacological blockade of TAAR1 with EPPTB significantly attenuates colitis severity by inhibiting trace amine-induced 5-HT signaling, reducing intestinal inflammation, and restoring gut barrier integrity. These findings highlight TAAR1 as a promising therapeutic target for IBD, offering a novel approach to mitigate disease progression by disrupting the microbiota-trace amine-5-HT axis. Further studies are warranted to validate these effects in additional IBD models and assess the translational potential of TAAR1 inhibitors in clinical settings.

## Funding

This work was kindly funded by 10.13039/501100003453Natural Science Foundation of Guangdong Province (2023A1515012475), Foundations of 10.13039/501100010877Shenzhen Science and Technology Innovation Committee (JCYJ20210324093810026) and Shenzhen high level hospital construction fund.

## CRediT authorship contribution statement

**Leyi Luo:** Formal analysis, Investigation, Methodology, Visualization. **Tong Zhang:** Formal analysis, Investigation, Methodology, Visualization. **Linlin Liang:** Investigation. **Weixin Chen:** Investigation. **Tao Wang:** Investigation. **Wenke Chen:** Resources. **Haitao Xiao:** Conceptualization, Funding acquisition, Project administration, Supervision. **Guangtao Zhang:** Conceptualization, Project administration, Supervision, Writing – original draft.

## Declaration of competing interest

The authors declare that they have no known competing financial interests or personal relationship that could have appeared to influence the work reported in this paper.

## Data Availability

All data supporting the findings of this study are available within the article.
