# Supplementary material for: The TAAR1 antagonist EPPTB ameliorates colitis via serotonin inhibition
Source: Biochem Biophys Rep. 2026 Jan 6;45:102432. doi: 10.1016/j.bbrep.2025.102432 (PMC12808517; doi:10.1016/j.bbrep.2025.102432)
Supplement: Multimedia component 1 [file mmc1.docx]

Figure 3C

GAPDH:CON,DSS,DSS+EPPTB


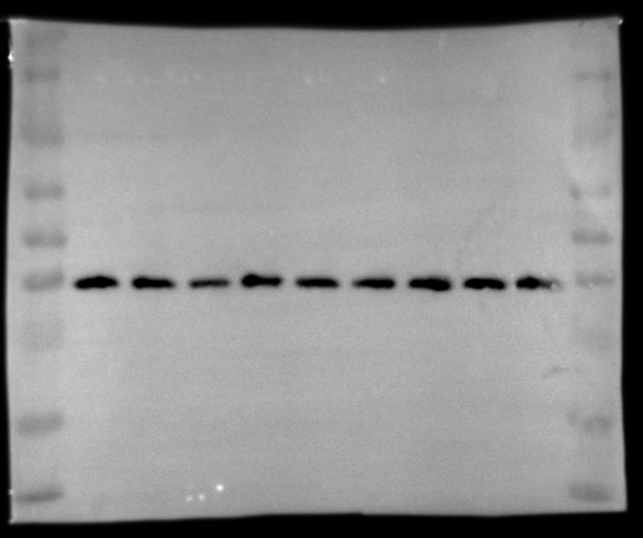


150kDa

100kDa

70kDa

50kDa

40kDa

35kDa

25kDa

20kDa

15kDa

GAPDH 36kDa

IκB-α:CON,DSS,DSS+EPPTB


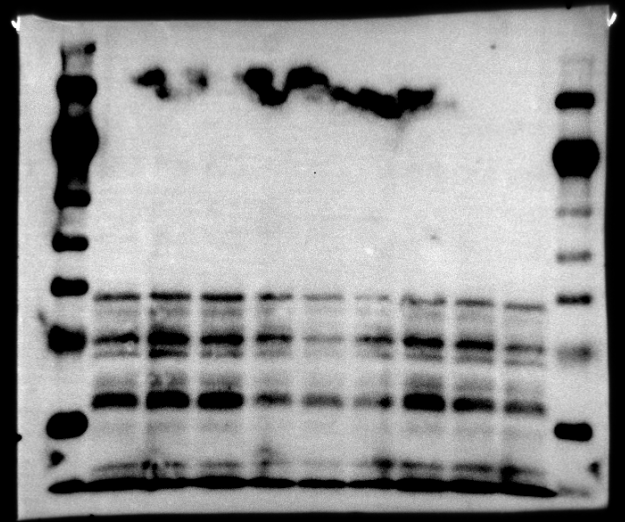


100kDa

150kDa

IκB-α 35kDa

70kDa

50kDa

40kDa

35kDa

25kDa

20kDa

15kDa

Figure 4C

GAPDH: CON,DSS,DSS+EPPTB


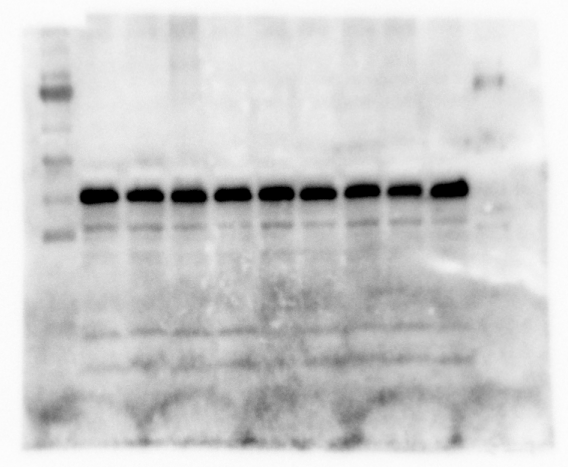


15kDa

GAPDH 36kDa

150kDa

100kDa

70kDa

50kDa

40kDa

35kDa

25kDa

20kDa

Claudin2: CON,DSS,DSS+EPPTB


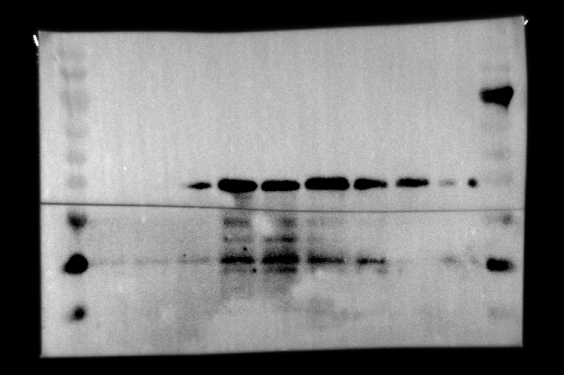


Claudin2 20kDa

100kDa

150kDa

70kDa

50kDa

40kDa

35kDa

25kDa

20kDa

15kDa

Occludin: CON,DSS,DSS+EPPTB


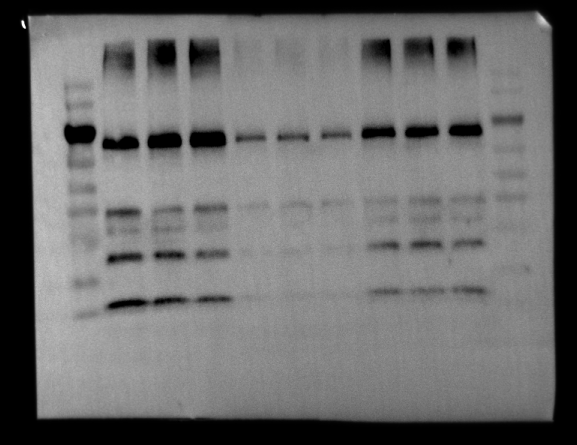


Occludin 65kDa

150kDa

100kDa

70kDa

50kDa

40kDa

35kDa

25kDa

20kDa

15kDa
