# Supplementary material for: The TAAR1 antagonist EPPTB ameliorates colitis via serotonin inhibition
Source: Biochem Biophys Rep. 2026 Jan 6;45:102432. doi: 10.1016/j.bbrep.2025.102432 (PMC12808517; doi:10.1016/j.bbrep.2025.102432)
Supplement: Multimedia component 2 [file mmc2.docx]

**Table S1 Corresponding clinical data of selected healthy subjects and UC patients**

| Characteristics | Healthy control (n = 15) | | Active UC (n = 20) |
| --- | --- | --- | --- |
| **Gender** | |  |  |
| Male | | 8 | 9 |
| Female | | 7 | 11 |
| **Age (years ± SD)** | | 49.47±9.13 | 49.80±9.30 |
| **Extent of disease** | |  |  |
| Extensive colitis | |  | 5 |
| Sided colitis | |  | 6 |
| Proctitis | |  | 9 |
| **Treatment** | |  |  |
| Amino salicylates | |  | 11 |
| Corticosteroids | |  | 8 |
| None | |  | 1 |
| **Endoscopy** | |  |  |
| Grade 0-1 | |  | 2 |
| Grade 2-3 | |  | 18 |
