# Supplementary material for: The TAAR1 antagonist EPPTB ameliorates colitis via serotonin inhibition
Source: Biochem Biophys Rep. 2026 Jan 6;45:102432. doi: 10.1016/j.bbrep.2025.102432 (PMC12808517; doi:10.1016/j.bbrep.2025.102432)
Supplement: Multimedia component 3 [file mmc3.docx]

**Table S2 Primers for experiments**

| Name of gene | Genetic sequence |
| --- | --- |
| mouse *GAPDH*-F | CATCACTGCCACCCAGAAGACTG |
| mouse *GAPDH*-R | ATGCCAGTGAGCTTCCCGTTCAG |
| mouse-*TNF-α*-F | GGGTGTTCATCCATTCTC |
| mouse-*TNF-α*-R | GGAAAGCCCATTTGAGT |
| mouse-*IL-1β*-F | GCAACTGTTCCTGAACTCAACT |
| mouse-*IL-1β*-R | ATCTTTTGGGGTCCGTCAACT |
| mouse-*IL-6*-F | CCAGTTGCCTTCTTGGGACT |
| mouse-*IL-6*-R | GTCTCCTCTCCGGACTTGTG |
| mouse-*ZO-1*-F | ACCCGAAACTGATGCTGTGGATAG |
| mouse-*ZO-1*-R | GCTGGCTGGCTGTACTGTGAG |
| mouse-*occludin*-F | AGGCAGCCTCGGTACAGCAG |
| mouse-*occludin*-R | AGGCAGCCTCGGTACAGCAG |
| mouse-*claudin-2*-F | AGCATTGTGACGGCGGTTGG |
| mouse*-claudin-2*-R | GGCAGCCTGGATGTCAGCAG |
| human-*TPH1*-F | TTCTGACCTGGACCATTGTGCC |
| human-*TPH1*-R | ACGGTAGACATTGTCTTTGAAGCC |
| human-*SLC6A4* -F | TCACAGTGCTCGGTTACATGGC |
| human-*SLC6A4* -R | GAAAGTGGACGCTGGCATGTTG |
| human *GAPDH*-F | GTCTCCTCTGACTTCAACAGCG |
| human *GAPDH*-R | ACCACCCTGTTGCTGTAGCCAA |
